# Healthy Public Policy Must Build Capacity: Analysing the Implementation of 3.2.2A, Australia's New Food Safety Standard

**DOI:** 10.1002/hpja.70206

**Published:** 2026-06-17

**Authors:** Jason Barnes, Harriet Whiley, Kirstin Ross

**Affiliations:** ^1^ School of Psychology and Public Health La Trobe University Bundoora Victoria Australia; ^2^ College of Science and Engineering Flinders University Bedford Park South Australia Australia

**Keywords:** food quality, food safety, government, health protection, policy, public health practice

## Abstract

**Issue Addressed:**

The integration of a new standard for food safety in Australia signifies a notable step toward national unity in the adoption of Hazard Analysis and Critical Control Point (HACCP) principles and contemporary food safety management tools for the food service and retail sector. The standard introduces key measures, including certification of food safety supervisors, food safety training requirements for food handlers, and means for substantiating control of food safety hazards throughout key food handling processes.

**Methods:**

This study explored the experiences and perspectives of local regulators regarding the implementation and feasibility of the new standard. Data were collected via an online survey and semi‐structured interviews with Environmental Health Practitioners across two Australian states.

**Results:**

Findings reveal that implementation of the standard varied between states, largely shaped by the degree of support provided by respective state authorities. Six key barriers to effective implementation emerged from participant accounts.

**Conclusions:**

In response, five targeted recommendations are proposed, grounded in established health promotion principles. Until these challenges are adequately addressed, the standard risks remaining ineffectual, limiting its capacity to enhance public health outcomes.

**So What?:**

The key to effective implementation and realisation of Standard 3.2.2A as healthy public policy lies within the central tenets of public health theory and practice.

## 1 Introduction

Australia's food safety regulatory system is widely recognised as a robust, internationally consistent and evidence‐informed health protection system [1]. Following reforms instigated in 1996, the food safety regulation framework in Australia has transformed in an effort to establish greater national consistency, safeguard trade and export opportunities, integrate evidence informed risk‐based approaches and enshrine outcomes‐based performance standards for food producers [2, 3, 4, 5, 6]. A bi‐national food standards code (the Code) devised in cooperation with New Zealand is a key product of these reforms. The Code provides important guidance in the form of a series of standards that underpin the broader food safety regulatory system in Australia, strongly reflecting the guiding values of the reforms; to wit, protection of public health and safety, promotion of consumer confidence, and preventing misleading conduct with regard to food [2, 6].

A notable characteristic of the Code is its lack of prescriptive requirements, instead reflecting performance standards and an outcome focus [3]. Thus, while placing the onus of producing safe and suitable food on food producers [3, 4, 5], it provides for flexibility in how food producers achieve conformity with the Code. As a set of national standards, the Code does not carry any legislative weight but rather is enshrined and enacted through jurisdictional (state‐based) legislation [6].

Although the reforms at the turn of the century had been enacted to overcome food regulation inconsistency between state and territory jurisdictions [2], food hygiene and food safety requirements still did not reflect complete uniformity across Australian jurisdictions [7]. A prominent divergence from other jurisdictions was that of the state of Victoria, with its risk classification framework for food businesses, a mandate for higher risk food businesses to maintain Food Safety Programs that reflected principles of HACCP planning or other quality assurance programs along with detailed substantiation components, and a requirement for the appointment and declaration of a Food Safety Supervisor within higher risk food businesses [7].

Recognising the merit of progressive food safety management devices in force in Victoria, and with a view to address the predominant contribution of the food service and retail business sector to foodborne illness [8, 9, 10], the development and integration of a new standard in the Code was proposed. The changes to the Code involved introduction of a new standard, 3.2.2A, to augment the existing food safety standards within the Code. This new standard contained elements reminiscent of some of the food safety management devices formerly mandated for food businesses handling unpackaged, potentially hazardous foods in Victoria. Thus, Standard 3.2.2A introduced three new food safety management duties for food service and food retail businesses handling unpackaged, potentially hazardous foods that are ready to eat [8, 11]. These are:

1. The requirement for a Food Safety Supervisor:
2. Food safety training for food handlers:
3. Substantiation of some critical control points and a pre‐requisite program.

While these new duties stopped short of the former Victorian requirements for a HACCP based food safety program, the new standard was a step toward the national adoption of food safety management tools that reflected HACCP principles and were customary of contemporary food safety regulatory policy.

### 1.1 Food Safety Supervision

Food safety supervision is a food safety policy approach aimed at establishing accountability in food production. Often deployed as a means of priming a positive food safety culture in food businesses [12], it establishes a level of authority in decisions and management of food safety, while also positioning a figure within the food business structure that can support training, model positive behaviours and establish communication and trust with food handlers within the business [13]. Behavioural integrity and food safety commitment demonstrated by food safety supervisors has been associated with a reduction in food safety risk and fostering safer food handling practices within food businesses [13], while higher levels of education and training of senior associates within food businesses have been shown to correlate with food business employees receiving food safety training [14]. The level of commitment to food safety by senior food business associates has also been associated with lower levels of microbiological contamination within their food premises [15]. While some food businesses within the service and retail sector with a trained and certified food safety supervisor have been shown to have fewer critical non‐compliances than those without, this is not representative of all types of food businesses within the sector [16] and in some instances has not been demonstrated to influence compliance status at all [17]. Nonetheless, the impact of food safety supervisors within a food business is likely determined by various personal and social factors such as manager effectiveness, understanding of role, attitudes, subjective norms and behavioural control [12, 18].

### 1.2 Food Safety Training for Food Handlers

Although the Code already required food handlers to hold the necessary skills and knowledge to handle food safely, Standard 3.2.2A introduced additional requirements for food handlers of in‐scope businesses. This comprised undertaking food safety training and refresher training every 5 years, or to demonstrate the adequacy of food handler skills and knowledge relevant to their role via suitable alternative means. Food safety training has been shown to improve food handling knowledge and practices [19, 20], and improve food safety culture in some cases [21]. Yet, while improved food safety knowledge is a common product of training, the translation of this knowledge into behavioural change, improvement of food handling practices and reduction of food safety risk often remain unfulfilled [22, 23, 24]. Thus, knowledge remains a central aspect for informing and facilitating safe food handling practices, albeit not the only determinant factor [25].

Hence, while knowledge is not the sole barrier, it is the primary barrier to the implementation of safe food handling procedures suggesting that the improvement of knowledge amongst food handlers is provident [25]. Baseline food safety knowledge amongst food handlers is highly variable and can be influenced by factors such as experience, age, education level and the quality of food safety training received [26, 27, 28, 29, 30]. The level of knowledge that participants attain from undertaking food safety training can be influenced by the sources of training, certification of the training, and their level of food handling experience, although the amount of time lapsed since undertaking training, in‐person or online format and the number of hours spent undertaking training tend not to influence knowledge attainment and retention [21, 31]. Despite the variability in the level of food safety knowledge amongst food handlers, the utility and ability to apply this knowledge may provide a more relevant metric [32, 33]. As demonstrated by the findings of Bolton et al. [24], while food handlers can be familiar with common microbiological pathogens associated with foodborne illness, they predominantly demonstrate a deficient understanding of the concept and application of HACCP and are widely unaware of relevant food safety laws and their ensuant legal obligations.

### 1.3 Substantiation

The final food safety management tool introduced with Standard 3.2.2A was the requirement for substantiation of control over some critical control points and one pre‐requisite program. Thus, records or other suitable evidence must be maintained for the following control points and prerequisite programs:

– Food Receipt—Temperature control.
– Food Storage—Temperature control.
– Food Processing—Pathogen reduction
– Food Processing—Temperature control for foods not undergoing pathogen reduction
– Food Cooling—Time and temperature control
– Food Reheating—Temperature [threshold]
– Food Display—Time and temperature control
– Food Transport—Temperature control.
– Cleaning and Sanitising [11]

While stopping short of the requirement for in‐scope businesses to prepare, implement and maintain a HACCP based food safety program, the requirement for substantiation does impel these food businesses to partially adopt some HACCP principles into their practices. This may signify a recognition of the unique modality of the food service and retail sector. Although integration of HACCP principles and planning is commonplace in food manufacturing, the integration into food service and retail businesses can be less cohesive and encounter some incompatibilities [34, 35].

### 1.4 Realisation of Standard 3.2.2A

The integration of the new Standard 3.2.2A signifies a notable step toward national unity in the adoption of HACCP principles and contemporary food safety management tools for the food service and retail sector. Yet, it is only through the effective and comprehensive implementation of this new standard that any benefits may be realised.

This research was aimed at understanding the experience and perspectives of frontline regulators in the implementation of Standard 3.2.2A. It sought to understand their views on the utility, the merit and the impact of the standard on regulatory practice and health protection.

## 2 Methods

Environmental health practitioners (EHPs) in South Australia and Victoria were invited to respond to an online survey via a weblink distributed through member emails and newsletters from environmental health peak bodies in those states. The online survey (Figure S1) was constructed and hosted using Qualtrics and comprised both closed and open ended questions about implementation of Standard 3.2.2A.

Survey data were analysed using the following methods:

1. Nominal survey data were analysed using basic descriptive statistics, namely frequency counts.
2. Free‐text responses were subjected to qualitative thematic analysis. Responses were coded inductively and assigned to themes.
3. Nominal data were then cross‐tabulated against variables of state and experience. Pearson's chi‐square analysis was performed using the Monte‐Carlo method. Cramer's V was also calculated to establish effect size, accompanied by bootstrapping to 5000 samples to inform confidence intervals of Cramer's V. Adjusted residuals were also calculated to identify the primary contributors to significant chi‐square results. All calculations were performed using IBM SPSS Statistics [36] and Microsoft Excel Version 2405.

Survey respondents were invited to register their interest in participating in further research via the online survey. Semi‐structured interviews were then carried out with 16 EHPs exploring their perceptions and experiences regarding the implementation of Standard 3.2.2A. Interview transcripts were analysed using a reflexive thematic analysis approach [37]. Researchers separately performed inductive coding followed by grouping of codes into wider themes. Themes were then refined through discussion and collaboration between researchers to establish their accuracy. This research received approval from the Flinders University Human Research and Ethics Committee (Ref #5058).

## 3 Results

**FIGURE 1:**
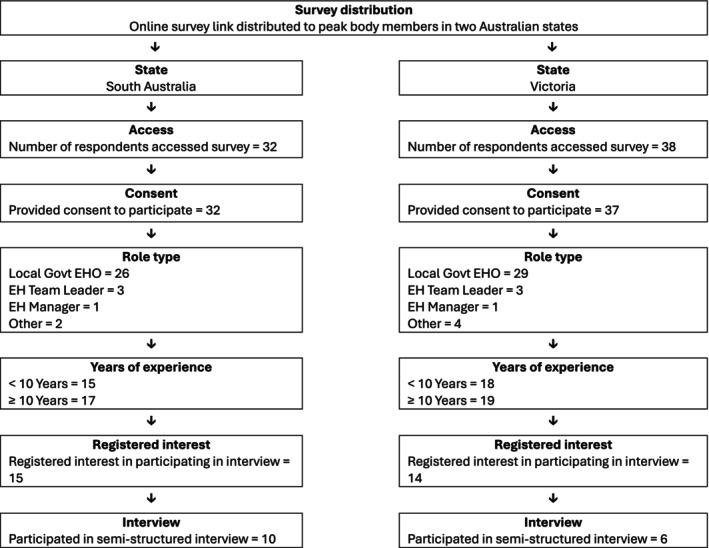
Research process and participant demographics.

A combined total of 69 respondents completed the online survey across South Australia and Victoria. A majority of respondents were Environmental Health Officers (EHOs) employed in Local Government. One person did not complete the consent form so their data were excluded. Approximately half of respondents had 10 or more years of experience in food safety regulation from both jurisdictions. A further 16 respondents also participated in semi‐structured interviews. Although the sample size of 69 is small, it represents just under 5% of EHOs employed across both states [38]. A diagram summarising survey and interview participation is provided in Figure 1.

### 3.1 Implementation and Perceived Impact of Standard 3.2.2A

**FIGURE 2:**
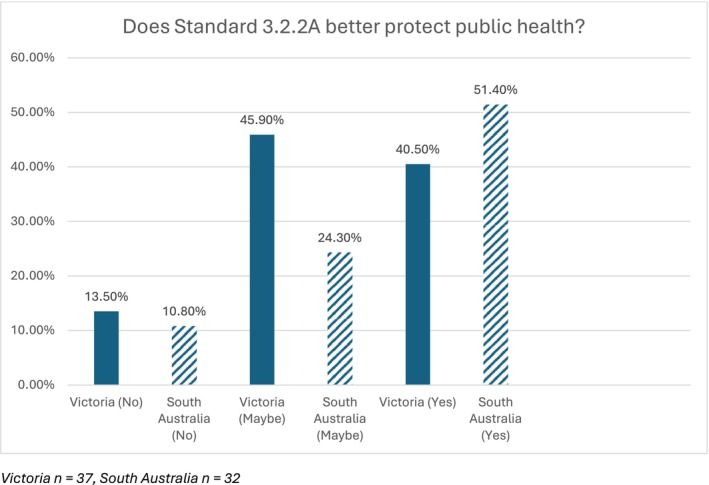
Does Standard 3.2.2A better protect public health?

Survey respondents were asked about their perceptions of whether Standard 3.2.2A stands to better protect public health. The frequency of responses are provided in Figure 2.

While 49% of respondents perceived the standard to better protect public health, 13% of respondents did not. Respondents were then provided an opportunity to describe the reason for their answer via a free‐text field. These data were thematically analysed for respondents that had indicated ‘no’ to the Standard better protecting public health. Seven themes were identified as barriers to Standard 3.2.2A protecting public health. The seven barriers identified were:

- Regulatory overreach
- Ineffectiveness of traditional compliance.
- Superficial training and knowledge deficiencies
- Insufficient changes to improve food handling practices
- Regulatory activities constrained by the need for building awareness of new requirements
- Superficial metrics are being used in demonstrating compliance
- Lack of tangible outcomes

**FIGURE 3:**
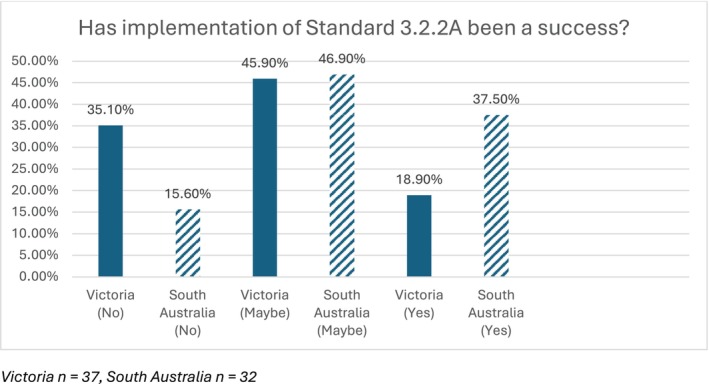
Has implementation of Standard 3.2.2A been a success?

Respondents were then asked if they perceived the implementation of Standard 3.2.2A to have been a success. The frequency of responses are shown in Figure 3.

The majority of respondents indicated uncertainty on whether implementation had been a success with 46% responding ‘maybe’. A further 26% of respondents indicated that the implementation of Standard 3.2.2A had not been a success. Respondents were again provided an opportunity to describe the reason for their answer via a free‐text field. These data were thematically analysed for respondents that had indicated ‘no’ to the implementation having been a success. Seven themes were identified as barriers to successful implementation of Standard 3.2.2A. Detailed responses provided by some participants were found to describe multiple barriers. The seven barriers identified were:

- Poor communication and rollout.
- Lack of clarity and usability in tools.
- Training and monitoring challenges
- Inconsistent implementation and guidance
- Impact on small business
- Misalignment between policy intent and outcome.
- Equity and accessibility issues

### 3.2 Implementation of Standard 3.2.2A

**FIGURE 4:**
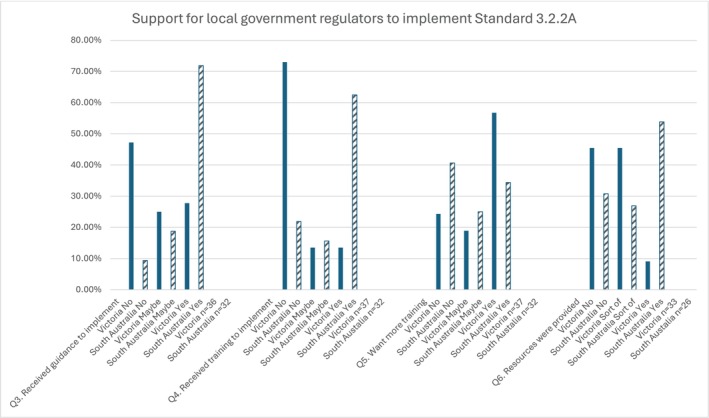
Support for local government regulators to implement Standard 3.2.2.

Survey respondents were then asked a series of questions about the support they received when implementing Standard 3.2.2A. Reflecting the nature of the food safety regulatory framework in place in Australia, state government authorities are largely responsible for facilitating integration of the new standard by making any necessary adjustments to food safety legislation, and acting as a point of centralised guidance and oversight of implementation for local government regulators. While the majority of survey respondents reported receiving guidance on the implementation of Standard 3.2.2A, most did not receive training or the necessary resources for implementation. The majority of respondents reported that they wanted to receive more training to support their implementation of the standard. The frequency of responses to these questions is provided in Figure 4.

When survey responses were separated between the two state jurisdictions, clear differences emerged. Specifically, while South Australian local government regulators largely reported receiving adequate support from their state government authority to facilitate implementation of Standard 3.2.2A, Victorian local government regulators did not receive the same level of support from their state authority as shown in Table 1.

**TABLE 1:** Statistically significant relationships with states and support for implementation of Standard 3.2.2A.

Independent variable	Dependent variable	*χ* ^2^	ɗʃ	*ρ*‐value (monte carlo)	Lower 95% confidence interval	Upper 95% confidence interval	Sample size (*N*)	Cramer's V	Lower 95% confidence interval	Upper 95% confidence interval	Minimum adjusted residual	Variable	Maximum adjusted residual	Variable
State	Received guidance	16.244	3	< 0.001	< 0.001	0.001	69	0.485	0.3	0.683	−3.7	Victoria–Yes	3.7	South Australia–Yes
State	Received training	20.51	2	< 0.001	< 0.001	< 0.001	69	0.545	0.367	0.74	−4.2	South Australia–No Victoria–Yes	4.2	South Australia–Yes Victoria–No
State	Resources provided	12.001	3	0.006	0.005	0.008	69	0.417	0.245	0.634	−3.4	Victoria–Yes	3.4	South Australia–Yes

### 3.3 Semi‐Structured Interviews

To gather more detailed accounts of local government regulators' experiences in implementing Standard 3.2.2A, 16 semi‐structured interviews were conducted. The following themes emerged through inductive thematic analysis of interview transcripts.

#### 3.3.1 Theme 1: Uniformity

Most interview participants noted their concerns about a perceived lack of consistency between local regulators in the implementation of Standard 3.2.2A. When these concerns were explored in greater depth, a lack of centralised guidance and regulatory oversight by the state authority, particularly in Victoria, was primarily cited as the cause. Participants perceived that a lack of communication, training and resources being provided by the state authority was necessitating local government regulators to undertake their own interpretation of the standard, devise regulatory frames, communicate changes and requirements to food businesses and to strategize rollout objectives and priorities on their own. Accordingly, this lack of centralised oversight and stewardship left implementation of Standard 3.2.2A liable to inconsistency and fragmentation:

“All 79 councils kind of fended for themselves to try and figure this out… Everyone's done it a little bit differently… because there's this inconsistency, how effective this new standard is, really is compromised…That shouldn't be put onto 79 councils to figure it out on their own.” Victorian participant 4.

“We are…probably not consistent with how others across the state are interpreting [3.2.2A] because it doesn't say you must have records.” South Australian participant 1.

#### 3.3.2 Theme 2: Training

While most interview participants agreed that the requirement for regular food safety training for food safety supervisors and food handlers at in‐scope food businesses signified positive progress, many were yet to identify improvement in food safety knowledge or food handling practices at food businesses. Some highlighted that the deadline for training completion was still pending at the time of the interview. Others expressed that training accessibility presented a barrier to some food businesses, particularly those requiring multilingual services. Yet some participants went further to challenge the core pedagogies underpinning food safety training programmes available to food businesses, describing them as inadequate for imparting practical knowledge and skills, and ineffectual in motivating behaviour change:

“Training devolves over time… training organisations have a way of devolving it into the simplest application and money‐making technique… The actual learnings from that education become problematic… or non‐existent in some cases… You can get through [training courses] without learning a damn thing… There's no effort to build a base of understanding” Victorian participant 5.

“X and X [EHOs employed at council‐names removed] did some [of the] training and what they were being taught was just ridiculous” South Australian participant 1.

“A lot of them [participants in food safety training] are just saying [training] is complete and utter waste of time” South Australian participant 11.

#### 3.3.3 Theme 3: Resources

Most participants saw a need for more resources to be made available to support food businesses implementing Standard 3.2.2A. Some respondents expressed their concern at the complexity and interpretability of materials that had been produced by some state authorities, while others remarked on the overall lack of materials available to food businesses. A distinct resource gap for food businesses identified by many participants was the lack of templates and lists of registered training providers. While South Australian participants saw value in record templates like those that were historically available in Victoria as part of food safety program templates, Victorian respondents expressed their discontent at the withdrawal of these food safety program templates during the state's transition to Standard 3.2.2A. The templates were recognised for their convenience, consistency, customisability, compliance standardisation and multilingual functionality:

“FoodSmart [food safety program template] has recently gone offline. It's no longer being provided as an option for us to use. One of the benefits of FoodSmart was that again, it was free to do, it was really specific to the business and it was available in other languages… Now that that's offline … sometimes it can be very difficult for [food businesses] to seek the information they need.” Victorian participant 3.

“We didn't have the resources…people were looking for the resources [from SA Health], but were then getting referred back to us.” South Australian participant 9.

#### 3.3.4 Theme 4: Regulation

Some respondents reported that the implementation of Standard 3.2.2A generated additional administrative burdens for regulators. These included the additional time required to assess compliance or to explain compliance obligations to food businesses during food safety inspections, as well as the challenge of tracking compliance with food safety supervision and training requirements. Some noted how this was exacerbated by the transient nature of food business employees throughout the industry and the lack of robust databases capable of recording training completion and expiry. Yet this burden was not experienced universally and a diversity of views were reported amongst participants, with some reporting high impost and others reporting negligible impact. Some respondents also described challenges arising from the incompatibility of Standard 3.2.2A with food safety law, highlighting the difficulties of translating an outcomes‐based standard into a compliance‐centric legal framework. This incompatibility was resulting in ambiguous compliance metrics that were driving uncertainty amongst regulators and food businesses:

“What does compliance look like… what evidence do we need to gather?… We had to be creative about how we administer this and implement in an existing regulatory framework… how do we adapt all our templates or methodologies?… It took weeks to do this… trying to still operate the unit with day‐to‐day stuff.” Victorian participant 4

“I can't see it [time requirement] getting less 'cause you still have to check [training completion]. The issue is that a lot of the places have higher turnover and staff, so you're going to have that transient workforce. So what we see on inspection one might not be inspection two.” South Australian participant 9.

#### 3.3.5 Theme 5: Consistency

All interview participants agreed that they saw great value in the national consistency established by introducing Standard 3.2.2A. Furthermore, there was a broad consensus amongst participants that the food safety management tools introduced with Standard 3.2.2A would positively contribute to food safety. Yet, most Victorian participants identified different aspects of the new food safety regulatory approach established with Standard 3.2.2A as a regression from the former food safety mechanisms in place in the state:

“In Victoria we started quite high and we've dropped down… other states started quite low and they've come up.” Victorian participant 3.

Some participants identified the legislative reform as regressive:

“the Victorian legislative reform was almost like regressing us backwards from being so progressive… The regulations have become a lot more convoluted… the effectiveness of the new standard is compromised.” Victorian participant 4.

While others identified regulatory backtracking as an inhibitor:

“They took that requirement away. Now, 12 months later, we've come back to [food businesses] and said… you basically need to do what you were doing before…The ones that always resisted it have sort of dug their heels in a little bit further now.” Victorian participant 1.

Other participants noted that the ambiguity introduced by Standard 3.2.2A led to uncertainty on the requirements for compliance:

“There's still an awful lot of that… do we need a food safety program for this or if they do this, does that exempt them?” Victorian participant 6.

Other participants identified that Standard 3.2.2A had exacerbated regulatory gaps:

“You have businesses that make allergen‐free claims… but they don't have that requirement to substantiate their practices.” Victorian participant 2.

#### 3.3.6 Theme 6: Engagement

Respondents reported the challenges and uncertainty faced by in‐scope food businesses with the introduction of Standard 3.2.2A. Some remarked on the distinct lack of communication and information that food businesses had received about the introduction of Standard 3.2.2A, while others noted the complexity of resources and materials precluding food businesses from utilising them. Many participants recognised the confusion and uncertainty that had developed amongst food businesses, particularly in terms of what they needed to do to meet the requirements of Standard 3.2.2A:

“Businesses have been really confused about what they're required to do” Victorian participant 2.

“Just considering the difficulties in implementing it when you're sort of chopping and changing all the time, it just makes it really difficult for businesses to understand what's required of them.” Victorian participant 1.

“A lot of them [food handlers] are a bit vague on the requirements.” South Australian participant 6.

This level of uncertainty amongst food businesses was observed by some participants to be leading to a perpetuation of standards in food businesses with no meaningful change:

“The businesses that had [good practices] already have continued on and the ones that didn't still don't.” Victorian participant 2.

## 4 Discussion

Introduction of Standard 3.2.2A in all Australian jurisdictions signifies a progressive step for food safety in Australia. The Standard introduces three key food safety tools for in‐scope food businesses, grounded in HACCP principles and aligned with industry best practices. These food safety tools are novel in most Australian jurisdictions and by introducing them through the national Food Standards Code, it aims for regulatory standardisation across Australia. This research investigated the experiences of local regulators in two Australian states when facilitating implementation of the new Standard. The experiences of local regulators were found to differ between the two states and this was largely influenced by the level of support provided by state authorities. Six key barriers to facilitating implementation of the new Standard were identified from the accounts of local regulators captured through an online survey and semi‐structured interviews. A health promotion lens has been applied in search of pragmatic solutions to overcome these barriers.

### 4.1 Recommendation 1: Position Capacity Building as a Central Intent of the Standard

The conceptualisation and implementation approach for this new Standard is reminiscent of most food safety policies recently established in Australia. Hence, the Standard forms part of the national Food Standards Code that is enshrined and empowered by legislation in each state jurisdiction [6, 39]. This legislative framework transforms the Standard from a set of food safety objectives into compliance obligations for duty holders. The point of difference for this new Standard from other elements of the Code, however, is the inclusion of tools that hold the potential to build capacity for food safety in the food service and retail sector. Viewing Food Safety Standards and their implementation outside of an exclusively regulatory lens may provide an opportunity to strengthen the capability and capacity of industry to produce safe food. Although the content of the Standard would likely remain static with these recommended changes, the shift in its conceptualisation would prime it to yield far greater value to all stakeholders.

### 4.2 Recommendation 2: Build Capacity Within Industry by Enabling Individuals to Develop [the Right] Personal Skills

The primary mechanism for this Standard to build capacity within the food service and retail industry is through food safety training and certification. Yet, as respondents highlight, the primary barrier to realising this benefit is the quality and design of food safety training systems currently in place in Australia. The issue is not about the need for longer training or refresher training, as these are unlikely to significantly improve food handler knowledge and practices [31], but rather the issue lies in the structure of learning, knowledge and skill development employed by these programmes. Food safety training in Australia is commonly delivered as competency‐based training [40, 41, 42]. It is an atheoretical exchange of facts, often delivered over a short period of time through a traditionalist pedagogical format. Learning is then examined by participants repeating those facts communicated or actions demonstrated to them [40]. This approach to teaching and learning does not adequately equip participants to minimise food safety risk because the knowledge and skills that are transferred to participants are irrelative to the causation and prevention of foodborne illness.

In order for food safety training to have a meaningful impact, it must instead be designed to build the capacity of trainees to independently identify and solve food safety problems [43, 44, 45]. An enhanced approach may adopt a more andragogical orientation that centres on problem‐solving and is supported by interactive technologies. This approach to teaching and learning may require greater investment from trainers and participants, particularly as participants develop skills in critical thinking, research and analysis. However, collaboration between food safety authorities, the food service and retail industry and the education and training sector to revise food safety training standards in Australia could lead to significant gains in food safety capability and capacity for the food service and retail sector and throughout the broader Australian community.

In realising this potential for redevised food safety training, it is important to recognise the true nature of the value it presents and to view it within the bounds of reasonable expectation. While it is likely to improve food safety literacy within the food service and retail sector, training does not represent a silver bullet to address poor food handling practices. Knowledge is not the exclusive determinant of behaviour [46], and thus it is unreasonable to expect that improvement in food safety knowledge will overcome all cases of unsatisfactory food handling.

### 4.3 Recommendation 3: Build Healthy Public Policy Through Legislative Reform

The legislative frameworks used to empower the Standard were another barrier identified by local regulators to facilitating implementation of the Standard. The Standard adopts an outcomes‐based approach, specifying the objectives to be achieved by food businesses rather than prescribing specific actions or duties for food businesses on how they must reach these outcomes [3, 4, 5]. Thus, outcomes‐based policy provides greater flexibility and discretion for food businesses to determine how they meet requirements. Conversely, legislative frameworks for food safety across Australia remain largely compliance‐centric, leaning toward a command‐and‐control paradigm [3]. This dilemma is not exclusive to Australia, being observed in other international jurisdictions attempting to implement risk‐based approaches [47]. Hence, regulators experience an incompatibility between the outcomes‐based approach of the Standard and the compliance‐centric ideology of the legislation through which it is enacted.

To address this incompatibility, a refinement of legislative frameworks across state jurisdictions may be beneficial, with greater incorporation of outcomes‐based, risk‐assessment principles. While this would entail some additional operational demand on state authorities, such adjustments could enhance alignment with Standard 3.2.2A without requiring fundamental structural change. More outcome‐focused and risk‐informed frameworks can support local regulators to draw on data and risk assessment in shaping their food safety assessments and determination of compliance, as well as to utilise existing legal tools more effectively to support public health outcomes and in doing so, contribute to a more holistic implementation of Standard 3.2.2A and the broader Food Standards Code.

Minor adjustments to registration structures currently established in food law also provides further opportunities to leverage Standard 3.2.2A in building healthy public policy and fostering supportive environments. Currently, food safety legislation across Australian jurisdictions imposes regulatory requirements through registration mechanisms linked to food premises or assets, rather than to individual persons. In contrast, Standard 3.2.2A introduces roles and responsibilities for individuals with its requirements for training and certification of food handlers and food safety supervisors [11]. Due to the highly transient nature of the food service and retail workforce [48], maintaining accurate training and certification records is inefficient when linked solely to premises registration. However, a modification to legislative frameworks to introduce individual licensing mechanisms and a licence database in accompaniment to premises registration could help overcome these data maintenance issues and create opportunities for food handlers. An individual food handler licensing scheme reminiscent of emergent micro‐credentialing systems would contribute to supportive environments by empowering individuals to develop and validate their skills, gain formal recognition, and pursue employment opportunities more freely within the food service and retail sector [49].

### 4.4 Recommendation 4: Adopt an Asset‐Based Approach

The food safety management tools introduced by Standard 3.2.2A are not entirely new across all Australian jurisdictions. While they do not directly replicate the food safety requirements historically used in Victoria, they reflect key elements of that system [7]. Local regulators have noted a lack of uniformity, resource gaps, and challenges arising from the loss of supporting materials and mechanisms like those previously available in Victoria.

This is likely due to the conceptualisation of the Standard and its implementation through a deficit paradigm, where gaps and needs are identified as a focus for development effort. Such an approach is common to the regulatory domain, although asset‐based development or appreciative inquiry are certainly growing in popularity across the broader domain of public health practice and community development [50]. Asset‐based development is an approach to development that identifies and leverages strengths with and within the community to drive outcomes [50, 51, 52]. Thus, assets or strengths may take the form of physical, human, social, financial or political capital relative to a community within a developmental context [53]. By adopting an asset‐based approach to implementing Standard 3.2.2A, state authorities in partnership with stakeholders can identify and leverage valuable resources and knowledge developed and refined through the former Victorian system. These assets could be adapted and leveraged to support both food businesses and regulators in implementing the new Standard. This approach also presents an opportunity for co‐design with local regulators and food businesses, allowing the Victorian templates and materials to be reshaped into contemporary, user‐responsive resources.

Importantly, however, an asset‐based approach should also recognise the true sources of value in these materials. Although Victorian local regulators have perceived Victoria's progressiveness in food safety regulatory systems to have been curbed with the introduction of Standard 3.2.2A, rates of foodborne illness in Victoria while the former regulatory systems were in place remained consistent with other states that lacked these food safety requirements and materials [54, 55, 56, 57, 58, 59, 60, 61, 62, 63, 64]. This suggests that while Victoria's regulatory tools were comprehensive, their primary value was not in preventing foodborne illness, but in supporting administrative efficiency for food businesses and streamlining data collection and review for regulators.

### 4.5 Recommendation 5: Strengthen Community Action Through Participation and Collaboration

Strengthening community action through participation and collaboration offers the most pivotal aspect to supporting comprehensive and effective implementation of Standard 3.2.2As demonstrated by the findings, limited engagement with local regulators and industry in implementation, development and resourcing of the standard resulted in fragmentation of approaches and increased pressure on regulatory resources. By engaging local regulators in the codesign of materials and resources for regulators, legislative reform, and evaluation and future revisions of the Standard, it provides an opportunity to foster uniformity and address resource gaps by meeting the needs of end users [65, 66]. By engaging with food businesses in the codesign of resources and materials for food businesses, establishing new standards for food safety training, evaluation and future revisions of the Standard, and devising a community led implementation strategy for the Standard, it provides an opportunity to produce resources and training standards that are relevant and meet the needs of end users, and fosters ownership and empowerment to drive a grass‐roots, community led implementation of the Standard [67, 68].

### 4.6 Strengths and Limitations

The limited sample size obtained through the online survey used to inform this research may constrain the generalizability of findings, particularly regarding the experiences of local regulators implementing Standard 3.2.2A beyond South Australia and Victoria. However, the 16 subsequent semi‐structured interviews reached data saturation and demonstrated robust triangulation between the two data sets, despite one being nested within the other. The aim of this research was to explore the experiences and perspectives of local regulators in implementing Standard 3.2.2A. This was achieved by capturing their insights into the utility, merit, and impact of the standard on regulatory practice and health protection. The value inherent in this paper spans from recommendations on how the identified barriers to implementation of Standard 3.2.2A may be overcome, providing for this Standard to be realised as healthy public policy. This research also provides a valuable foundation for future research that examines enforcement responses applied for non‐compliance with Standard 3.2.2A.

## 5 Conclusion

Standard 3.2.2A represents a well‐designed food safety policy and marks a significant advancement for Australia, particularly in progressing toward national regulatory standardisation. However, its implementation has varied across states, largely shaped by the degree of support provided by respective state authorities. This research identified six key barriers hindering effective implementation. In response, five targeted recommendations have been proposed, grounded in established health promotion principles. Until these challenges are adequately addressed, the standard risks remaining ineffectual, limiting its capacity to enhance public health outcomes. The key to effective implementation and realisation of Standard 3.2.2A as healthy public policy lies within the central tenets of public health theory and practice.

## Author Contributions

J.B.: conceptualisation, data curation, formal analysis, investigation, writing – original draft; H.W.: conceptualisation, funding acquisition, investigation, methodology, writing – review and editing. K.R.: conceptualisation, data curation, funding, investigation, visualisation, writing – review and editing.

## Funding

This work was supported by the Local Government Association South Australia #2024.53.

## Ethics Statement

This research received approval from the Flinders University Human Research and Ethics Committee (Ref #5058).

## Conflicts of Interest

The authors declare no conflicts of interest.

## Supporting information


**Figure S1:** Online survey.

## Data Availability

The data that support the findings of this study are available on request from the corresponding author. The data are not publicly available due to privacy or ethical restrictions.
