# Supplementary material for: Healthy Public Policy Must Build Capacity: Analysing the Implementation of 3.2.2A, Australia's New Food Safety Standard
Source: Health Promot J Austr. 2026 Jun 17;37(3):e70206. doi: 10.1002/hpja.70206 (PMC13275183; doi:10.1002/hpja.70206)
Supplement: Supplementary file 1 — Figure S1: Online survey. [file HPJA-37-0-s001.docx]

Supplementary Figure 1. Online Survey

How inspectors in South Australia / Victoria perceive the new Food Safety Standard 3.2.2A

Start of Block: Default Question Block

Information Sheet and Consent form

________________________________________________________________

Do you consent to participating in this online survey?

- Yes, I consent to participate (1)
- No, I do not wish to participate - just close this browser (2)

What is your current role?

- Local government EHO (1)
- Local government Environmental Health Team leader (2)
- Local government Environmental Health Manager (3)
- State Government - Vic DH (4)
- Other - please explain (5) __________________________________________________

How many years have you been working in Environmental Health?

- less than one year (1)
- 1 - 2 years (2)
- 2 - 5 years (3)
- 5 - 10 years (4)
- More than 10 years (5)

Thinking about the intent of the new Food Safety Standard 3.2.2A - overall, do you think it better protects public health?

- Yes (1)
- Maybe (2)
- No (3)

Would you like to provide more detail?

________________________________________________________________

Thinking about the implementation in Victoria of the new Food Safety Standard 3.2.2A - do you think it has been a success?

- Yes (1)
- Maybe (2)
- No (3)

Would you like to provide more detail?

________________________________________________________________

Thinking about the implementation of the new Food Safety Standard 3.2.2A - did you receive guidance that was adequate to implement the new standard?

- Yes (1)
- Maybe (2)
- No (3)

Would you like to provide more detail?

________________________________________________________________

Thinking about the implementation of the new Food Safety Standard 3.2.2A - did you receive any training to support the implemention?

- Yes (1)
- Maybe (2)
- No (3)

Would you like to provide more detail?

________________________________________________________________

On a scale of one to ten, how useful was the training that you received? (if you did more than one training session, please explain below in the "further detail" question)

|  | 0 | 1 | 2 | 3 | 4 | 5 | 6 | 7 | 8 | 9 | 10 |
| --- | --- | --- | --- | --- | --- | --- | --- | --- | --- | --- | --- |

| 0 is not useful at all, 10 is super useful (1) | 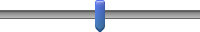 |
| --- | --- |

Would you like to provide more detail?

________________________________________________________________

Thinking about the implementation of the new Food Safety Standard 3.2.2A - would you like more training?

- Yes (1)
- Maybe (2)
- No (3)

Would you like to provide more detail?

________________________________________________________________

How much extra time do you think that the implementation of the Standard is taking you per food premises inspection? ( in minutes)

________________________________________________________________

Would you like to provide more detail?

________________________________________________________________

Are there resources that the implementation of the Standard needed or needs?

________________________________________________________________

Have these resources been provided for you?

- Yes (1)
- Sort of (2)
- No (3)

Would you like to provide more detail?

________________________________________________________________

What is working well?

________________________________________________________________

Would you like to provide more detail?

________________________________________________________________

What are the challenges?

________________________________________________________________

If you have had challenges, what things have you done to overcome them?

________________________________________________________________

Lastly, it would be useful for us to know a bit about how food proprietors felt the impacts have been to their business - can you comment?

________________________________________________________________

We would like to talk to you to explore your answers further - if you are willing to do this please type your email address below and one of the researchers will make contact:

________________________________________________________________

End of Block: Default Question Block
